# After one year in university; a robust decrease in autistic traits reporting among autistic students

**DOI:** 10.3389/fpsyt.2023.1146819

**Published:** 2023-07-03

**Authors:** Gil Zukerman, Gili Yahav, Ester Ben-Itzchak

**Affiliations:** ^1^Department of Communication Disorders, Ariel University, Ariel, Israel; ^2^Bruckner Center for Autism Research, Ariel University, Ariel, Israel

**Keywords:** autism traits, post-secondary education, anxiety symptoms, autism support programs, attention alterations

## Abstract

**Background:**

Previous research on autistic students enrolled in university support programs has reported moderate improvement in anxiety/depression or adaptive behavior. However, alterations in autistic traits have not been examined.

**Methods:**

This longitudinal study evaluated changes in university students’ autistic trait and state/trait anxiety levels. Participants were 24 neurotypically developed (ND) students with high levels of social anxiety symptoms (High SA), 30 ND students with low levels of SA symptoms (Low SA), and 41 autistic students (the primary focus of this study) residing with an ND peer student mentor as part of participating in the university’s integration support program. Autism spectrum quotient [AQ and State Trait Anxiety Inventory STAI] data were collected during the first semester of two consecutive academic years (T1, T2), as well as baseline (T1) levels of social anxiety, depression, and obsessive–compulsive symptoms.

**Results:**

Significant interaction between group and time was observed, denoting a sharp decrease (2.9 SD) from T1 to T2 in the overall autistic trait level among the autistic group (AQ “attention switching” subscale demonstrating the most robust decrease), and a moderate decrease (0.5 SD) among the high SA group. Only for the autistic students were more compulsive symptoms at T1 associated with a lesser decrease in AQ scores (T1-T2), which in turn was negatively correlated with their T1 year-end grade point average.

**Conclusion:**

The findings suggest that attending post-secondary education (while partaking in a support/transition program) is followed by a profound change of the individual’s subjective experience of autism, characterized by a sharp decline in the level of autistic traits, particularly attention switching. This change is independent of alterations in well-being indices, such as anxiety, that are known to characterize students attending university.

## 1. Introduction

Autism spectrum disorder (ASD) is a neurodevelopmental disorder characterized by social communication/interaction impairment and restricted/repetitive behaviors, interests, or activities [DSM 5, (1–3)]. Autism is estimated to occur in 1 in 44 people [U.S.; (4)] and approximately 67–70% have no intellectual disability (4, 5).

In recent years, individuals with autism are increasingly enrolling in higher education (6), the matriculation to which can be a complex experience. Indeed, autistic students attending university have reported elevated personal distress (6–10) along with high rates of anxiety and depressive symptoms, in particular social anxiety and obsessive–compulsive symptoms (11–15). The relatively high rates of comorbid psychiatric symptoms, specifically anxiety, may have a profound effect on their acclimation to university: previous studies have reported a negative association between anxiety (especially social anxiety and obsessive compulsive symptoms) on adaptive behavior and academic performance among young undergraduate autistic students (10, 14–16) as well as autistic children and adolescents (17, 18).

In light of the rapid increase in university students with autism (19), the necessity of developing appropriate support programs has been acknowledged (20, 21). However, few programs have been described in the literature, and efficacy research is even more scarce. The majority of the existing studies have focused on well-being and functional indices such as anxiety and depression (16, 22–25), satisfaction (25), self-efficacy (23), and adaptive behavior (16): moderate improvement in these well-being and functional indices has been reported. However, the possibility of change in autistic characteristics during university attendance in the context of support program participation has yet to be examined.

Higher education integration support programs may have a fundamental effect on autism trajectories and outcomes, as suggested by the effect of intensive intervention efforts during other developmental stages (usually early intervention among toddlers) that has resulted in reported gains in cognitive and adaptive functioning, as well as decreased ASD symptom severity (26–28). It has been suggested that use of multisensory and multidomain teaching approaches can alter core deficits in social-communication domains by providing recurring experiences that promote increased complexity in neural networks and connectivity (29, 30). Thus, it is highly relevant to study whether the experience of attending university (whether or not partaking in a specialized transition program) may serve as a window of opportunity to alter autism trajectories and enhance functioning later in life.

Support programs should also take into consideration the specific characteristics associated with autism. Autistic traits, defined as a set of personality characteristics, have been associated with the phenotypic expression of autism (31), and often include social skill impairment, a strong preference for routine, difficulties with attention switching, and impaired imagination (32). Significantly higher in autism (33), these traits are conceptualized as representing a normally distributed continuum of autistic features among the general population. The Autism Spectrum Quotient [AQ, (1)] is one of the most widely used quantitative measures of the levels of autistic traits reflecting the broader autistic phenotype spectrum (34). Several studies have examined the association between high levels of autistic traits, as measured by the AQ, and various factors. One line of research examined the association between AQ scores and mental health/personality factors, finding high levels of autistic traits among ND students associated with mental distress, particularly anxiety (35–38); this has led to the suggestion that AQ reporting is affected by the individual’s mental state (39). However, changes among autistic students may be less associated with anxiety alteration while other factors contributing to change may occur.

The current study’s primary aim, therefore, was to explore whether there is change in autistic students’ self-reported autistic traits during 1 year of attending university. The autistic students in our cohort were participating in the university’s integration program, which included facilitated social interaction with an ND peer-mentor with whom the vast majority of autistic students resided, as well as extensive focused opportunities for socializing via various program activities. Since social anxiety has been reported as the most frequent type of comorbid anxiety among autistic individuals (40, 41) and, like autism, it also significantly impacts social abilities (14, 42), ND students with high and low levels of social anxiety served as control groups.

We hypothesized that a year of participating in the university’s integration program would be followed by reports of lower levels of autistic traits. We also hypothesized that changes in levels of general anxiety over that year would be associated with changes in autistic traits. A secondary aim of the study was to examine whether reduction in AQ scores after one academic year would correlate with students’ baseline levels of psychiatric symptoms (social anxiety, depression, and obsessive–compulsive symptoms). We hypothesized that lower baseline levels of psychiatric symptoms would be associated with greater AQ reduction one academic year later. Previous study findings have demonstrated positive or negative associations (38, 43) between autistic traits and academic performance. Accordingly, we hypothesized that changes in the level of autistic traits and academic achievement (annual grade point average at the end of the academic year) would be significantly associated. However, no direction of that association was assumed.

## 2. Materials and methods

### 2.1. Participants

The study included 95 full-time undergraduate university students (11 females; *M*_age_ = 24.19 years, *SD* = 2.65), who were assigned to one of three groups: (1) Students diagnosed with autism spectrum disorder (Autism group; *n* = 41). (2) ND students with high social anxiety symptom levels (High SA; *n* = 24); and (3) ND students with low social anxiety symptom levels (Low SA; *n* = 30). As students with autism tend to gravitate toward science, technology, and mathematics fields (44), the vast majority of the autistic students were enrolled in exact sciences and engineering departments; accordingly, advertisements soliciting ND student participation in the study were mainly posted in exact sciences facilities.

Formal diagnoses for the autistic students were collected from the university integration program’s files (with participant consent) and the inclusion criteria consisted of an Autism Spectrum Disorder (ASD) diagnosis based on DSM-5 or ICD-10 criteria from a licensed neurologist/psychiatrist/psychologist. All Autism group participants had attended mainstream schools prior to attending university, had met standard university requirements for the department to which they were accepted, and were participating in the university’s integration program. They were quite independent and required no assistance in activities of daily living. Table 1 presents participant demographics, year-end Grade Point Average (GPAs), and psychiatric comorbidities (Low SA symptoms group participants were asked to specify any previous psychiatric diagnoses).

**Table 1:** Demographics and academic affiliation among the study groups.

	ASD (*n* = 41)	High SA (*n* = 24)	Low SA (*n* = 30)
Mean age (*SD*)*	23.46 (2.89)	24.33 (1.81)	25.07 (2.88)
Race (%)			
Caucasian	100%	100%	100%
Sex (%)(*n*)			
Female	4.87% (2)	8.33% (2)	23/33% (7)
Academic affiliation^a^ (*n*)			
Exact sciences	21	24	2
Engineering	5	0	8
Life sciences	1	0	0
Health sciences	0	0	3
Architecture	0	0	1
Communications	0	0	2
Humanities	4	0	9
Grade point average (*SD*) (year-end)	76.12 (18.11)	77.09 (11.39)	83.07 (7.03)
Psychiatric comorbidities^b^ (%)(*n*)			
ADHD*	19.5% (8)	–	10% (3)
Learning disability**	24.3% (10)	–	3% (1)
Anxiety disorder**	14.63% (6)	–	0
Depressive disorder	2.4% (1)	–	0
OCD*	9.7% (4)	–	0
Disruptive, impulse control, and conduct disorder	2.4% (1)	–	0
Personality disorder	2.4% (1)	–	0
Psychiatric medication (%)(*n*)	2.4% (1)	–	0

ADHD, attention deficit hyperactivity disorder; ASD, autism spectrum disorder; OCD, obsessive compulsive disorder; SA, social anxiety. ^a^Academic affiliation data available for 34/41 participants of the Autism group, 24/24 of the High SA group, and 29/30 of the Low SA group. ^b^Psychiatric comorbidities were excerpted from students’ files with their consent. *p < 0.05, **p < 0.01.

Participants from all three groups completed the Liebowitz Social Anxiety Scale [LSAS; (45)]. ND participants with LSAS scores ≥30 [considered the optimal cutoff signifying the presence of Social Anxiety Disorder; (46, 47)] were included in the high social anxiety symptom (High SA) group; those with scores <30 were included in the low social anxiety symptoms (Low SA) group. Significant group differences in age were observed, *F*(2, 94) = 3.26, *p* = 0.043. Post-hoc (Bonferroni) testing indicated that the Autism group was significantly younger than the Low SA group. Male predominance was observed, with significant difference observed between groups, χ^2^(2,95) = 6.09, *p* = 0.048.

### 2.2. Integration support program

The university’s integration program primarily included: (1) Mentoring each autistic student was matched with a ND student (peer–mentor). The vast majority (83%) of autistic students resided with their peer-mentor in the university dormitories for at least one academic year (in which the study took place), while the remaining students resided either at home with their parents (13%) or in rented apartments near the university (~4%). The autistic students regularly met with their mentors over the same period. Peer-mentors provided daily support to their mentees and encouraged social interaction. (2) Tutoring – the autistic students attended a weekly half-hour personal session with a program coordinator that included information and assistance regarding academic procedures. (3) Structured social activities - all autistic students and their mentors had once-weekly (~25 times per academic year) two-hour social events. Additionally, the autistic students attended separate lectures (e.g., topics such as sexuality, employment opportunities, etc.). Overall, autistic students spent about 3 h weekly on program-related activities.

### 2.3. Measures

#### 2.3.1. Autism spectrum quotient

The autism spectrum quotient (AQ) quantifies autistic traits among adults with average IQs (1), tapping into an individual’s agreement with 50 statements on a 4-point Likert scale. The AQ includes five subscales (Social Skills, Attention Switching, Attention to Detail, Communication, Imagination) and can be used as a screening tool for autism (48). Relatively good test–retest reliability and internal consistency have been reported (1).

#### 2.3.2. Liebowitz social anxiety scale

Assesses social interaction and performance anxiety by presenting 24 Likert scale items (range: 0–3) querying experienced fear and avoidance (49, 50). A total score, and fear and avoidance subscale scores, are generated (49). High reliability and validity have been reported for the self-report version used in this study (51).

#### 2.3.3. Beck depression inventory

A 21-item self-report measure assessing cognitive, affective, and behavioral outcomes of depression (52). High reliability and validity, expressed in high correlations with other depression-related self-rating scales (*r* = 0.66), have been reported (52, 53).

#### 2.3.4. Yale–Brown obsessive–compulsive scale II

Measures obsessive–compulsive disorder (OCD) symptom severity (54), yielding a total score (range: 0–40) and subscores for obsessions and compulsions (range: 0–20 for each). Moderate to high reliability and significant correlation with other OCD clinical symptom scales have been reported (55).

#### 2.3.5. State trait anxiety inventory

The state trait anxiety inventory (STAI) produces two scores: trait and state anxiety (2, 56). High internal consistency coefficients (0.86–0.95), acceptable validity and moderate validity were reported (2).

#### 2.3.6. Grade point average

The participants’ year-end grade point average was obtained from an official university-generated report. GPAs were in numeric values, ranging from 0 to 100 (Table 1).

### 2.4. Procedure

The study was approved by the university research ethics committee. All procedures were in accordance with ethical standards of the responsible committee on human experimentation (institutional and national) and the Helsinki Declaration (1975, revised in 2000). All participants provided signed informed consent to participate. The AQ, Beck depression inventory (BDI-II), LSAS, and STAI were self-completed a research assistant was available for questions, while the Yale–Brown Obsessive–Compulsive Scale II (Y-BOCS-II) was administered by a licensed clinical psychologist. Completion of the instruments took place in this order at the start of the 1^st^ academic year (T1 = Time 1), and over about 1.5 h. The AQ and STAI were also administered (in that order) at the start of the 2nd academic year (T2 = Time 2).

### 2.5. Data analysis

Analyses were conducted using SPSS version 25 (57). AQ total and subscale scores and year-end GPA were transformed to normal distribution in accordance with Templeton (57, 58). Transformed AQ scores and grade point averages were examined for normality, in accordance with Kim (59). Chi square analyses were conducted to assess group differences in sex and rates of previous psychiatric diagnoses (see Table 1). Univariate analyses were used to assess group differences in psychiatric symptoms levels (LSAS, BDI-II, Y-BOCS-II) at T1. To assess change over time for autism traits, AQ total score and AQ subscale scores (Social Skills, Attention Switching, Attention to Detail, Communication, Imagination) were entered as dependent variables in two separate analyses that included a 3 × 2 (3 groups × 2 time points) multivariate analysis of variance with repeated measures for time, and 3 × 2 doubly multivariate analysis of variance with repeated measures for time, respectively.

**Figure 1:**
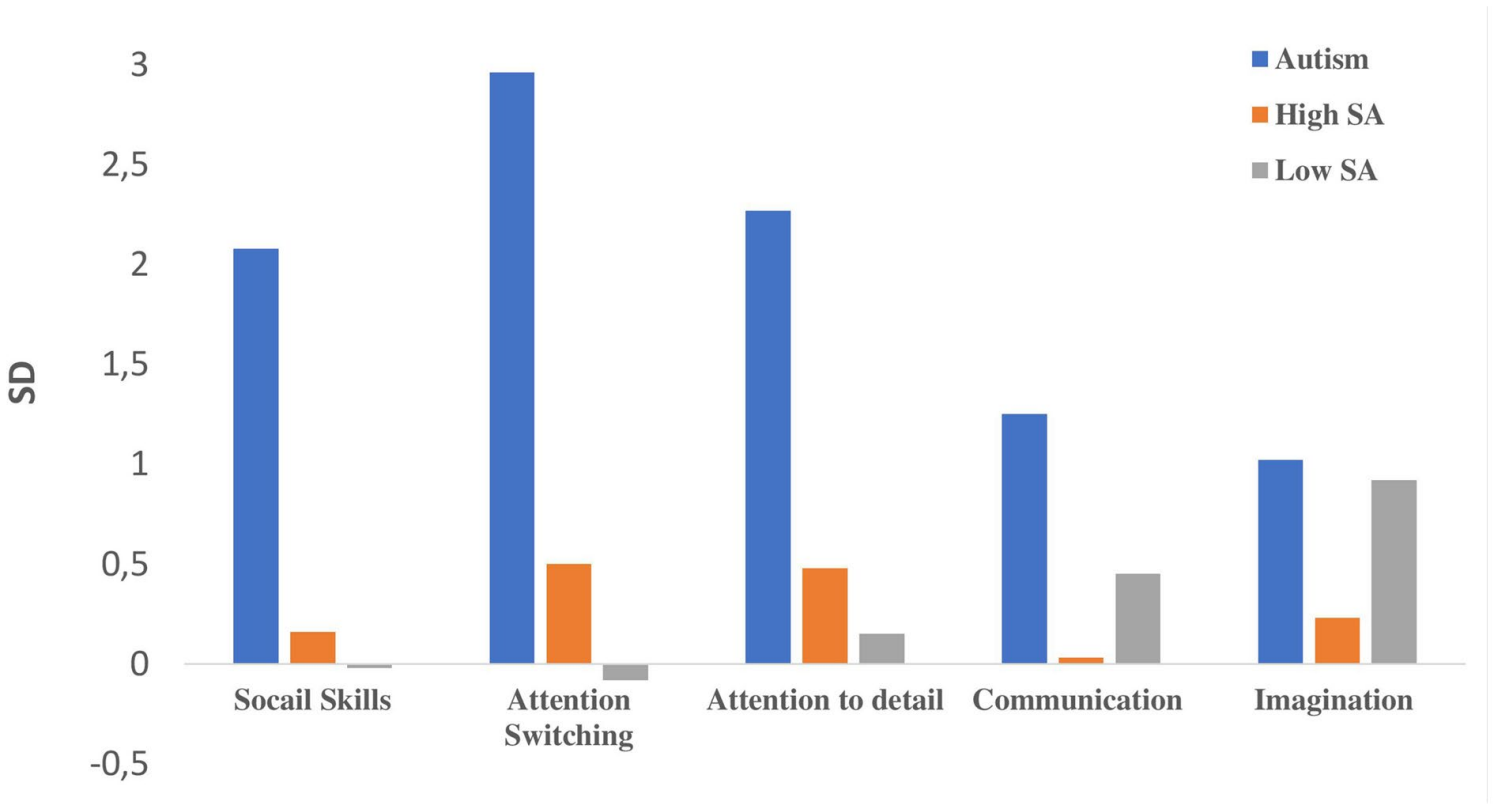
Reduction in AQ subscales scores (T1-T2) among the study’s groups.

All analyses were conducted with/without Sex and Age as covariates. We used SD’s taken from Baron-Cohen et al. (1) for presentation of the difference in AQ subscale scores (T2-T1) (see Figure 1). An additional MANOVA was conducted to assess change over time in anxiety levels (STAI State and Trait scores) among the study groups. Correlation analyses with False Discovery Rate corrections for multiple comparisons [FDR, (60)] were conducted between changes in AQ subscale scores (T1-T2), general anxiety (STAI T1-T2 trait and state subscales), and year-end GPAs.

Five linear regression analyses were conducted to examine the contribution of psychiatric symptoms to the variance in AQ subscale score change (T1-T2) over 1 year of university attendance. Sex and age were entered in the first step, ASD diagnosis (yes/no) and social anxiety symptom level (T1 LSAS total score) in the second step, depression symptom level (T1 BDI-II) and compulsion level (T1 Y-BOCS-II score) in the third step. The interactions of ASD Diagnosis (yes/no) with Depression level (ASD diagnosis* T1 BDI-II) and ASD Diagnosis and Compulsion level (ASD diagnosis*T1 Y-BOCS-II compulsion) were entered in the fourth step, in a stepwise manner. Due to the high correlation between obsession and compulsion scores (*r* = 0.84) only the latter was entered into the regression models. Continuous variables were standardized for regression analyses according to Dawson. All independent variables in the regression analysis were examined for multicollinearity.

## 3. Results

All the study’s transformed outcome variables (total and subscale AQ scores, GPA) were normally distributed. At T1, significant group differences were found in the social anxiety symptom levels (higher LSAS scores in the Autism and High SA groups compared to the Low SA group), as well as higher levels of depression symptoms (although BDI-II scores were in the subclinical range for all groups) and higher obsessive and compulsive symptoms (Y-BOCS-II scores) among the Autism group compared to the other groups (Table 2).

**Table 2:** Psychiatric symptoms among the study’s groups at T1.

	Autism (SD)	High SA (SD)	Low SA (SD)
LSAS total score**	45.54 (25.65)	54.43 (15.69)	19.15 (7.92)
BDI-II*	10.78 (7.89)	6.66 (5.76)	6.83 (6.31)
Y-BOCS-II obsession score**	7.60 (3.74)	3.08 (3.83)	3.13 (3.47)
Y-BOCS-II compulsion score**	7.36 (3.23)	2.04 (3.37)	2.56 (3.47)
			

LSAS, Liebowitz Social Anxiety Scale; BDI–II, Beck Depression Inventory II; Y-BOCS-II, Yale-Brown Obsessive–Compulsive Scale II. *p < 0.05, **p < 0.01.

### 3.1. AQ and STAI score change

#### 3.1.1. AQ total score change

The change (T1-T2) in AQ total score after one year of university attendance was examined for all groups. The 2 × 3 univariate ANOVA with repeated measures for time yielded a significant Group × Time interaction [*F*(2, 91) = 26.09, *p* < 0.00, η^2^_p_ = 0.36]. This effect remained significant when using Age and Sex as covariates. Separate analyses for each group yielded a significant Time effect for the Autism group [*F*(1, 40) = 103.62, *p* < 0.00, *η^2^_p_* = 0.72], (T1 *M* = 42.11, *SD* = 8.33; T2 *M* = 23.29, *SD* = 6.59) and for the High SA group, [*F*(1, 22) = 5.91, *p* = 0.02, η^2^_p_ = 0.21] (T1 *M* = 19.05, *SD* = 9.73; T2 *M* = 15.68, *SD* = 10.08). Lower AQ total scores were noted at the start of the second academic year as compared to the start of the first year, for both the Autism and High SA groups. The findings also indicate a significant Time effect [*F*(1, 91) = 63.63, *p* < 0.00, η^2^_p_ = 0.41, with lower AQ total scores at T2 (M = 17.51, *SE* = 0.75) compared to T1 (*M* = 26.13, *SE* = 0.03)]. Additionally, a significant Group effect was observed [*F*(2, 91) = 82.23, *p* < 0.00, η^2^ = 0.64], indicating significantly higher AQ scores among the Autism group (*M* = 32.70, *SD* = 7.46) compared to the High SA (*M* = 17.44, *SD* = 9.91) and Low SA (*M* = 15.32, *SD* = 6.76) groups. The Time effect was not significant when using Age as a covariate.

#### 3.1.2. AQ subscale change

For AQ subscale changes, the 2 × 3 MANOVA with repeated measures for time yielded a significant Group × Time interaction [*F*(10, 172) = 20.23, *p* < 0.00, η^2^_p_ = 0.54]. This effect remained significant even when using Age and Sex as covariates. Univariate tests indicated a significant interaction in all AQ subscale domains (*p* < 0.000 for all). Separate analysis for each group yielded a significant Time effect for the Autism group [*F*(5, 36) = 93.15, *p* < 0.00, η^2^_p_ = 0.92]. Univariate analyses for each separate AQ subscale indicated a significant Time effect for all subscales; a robust decrease in AQ scores was evident for all AQ subdomains following one academic year (see Table 3). In addition, a significant Time effect was noted for the High SA group [*F*(5, 18) = 3.78, *p* = 0.01, η^2^_p_ = 0.50], However, univariate analyses indicated that the decrease in AQ scores was significant only for the Attention Switching, Attention to Detail, and Imagination subscales (Table 3). A significant Time effect was also noted for the Low SA group [*F*(5, 25) = 5.44, *p* < 0.00, η^2^_p_ = 0.52], however univariate analyses revealed a significant decrease only for the Communication and Imagination subdomain scores (see Table 3).

**Table 3:** Time effects on AQ subscales in each of the study’s groups.

AQ subscale	Autism			High SA			Low SA			*F*(1, 40)	*p*	η^2^	*F*(1, 22)	*p*	η^2^	*F*(1, 29)	*p*	η^2^
Social skills	**87.87**	**0.00**	**0.68**	1.18	0.28	0.05	0.21	0.88	0.00
Attention switching	**401.47**	**0.00**	**0.90**	**12.28**	**0.00**	**0.35**	0.13	0.71	0.00
Attention to detail	**207.11**	**0.00**	**0.83**	**6.45**	**0.01**	**0.22**	0.77	0.37	0.02
Communication	**23.68**	**0.00**	**0.37**	0.08	0.77	0.00	**5.79**	**0.02**	**0.16**
Imagination	**26.61**	**0.00**	**0.40**	**11.15**	**0.00**	**0.33**	**24.12**	**0.00**	**0.45**

Bold values in table represent *p* <0.05.

**Figure 2:**
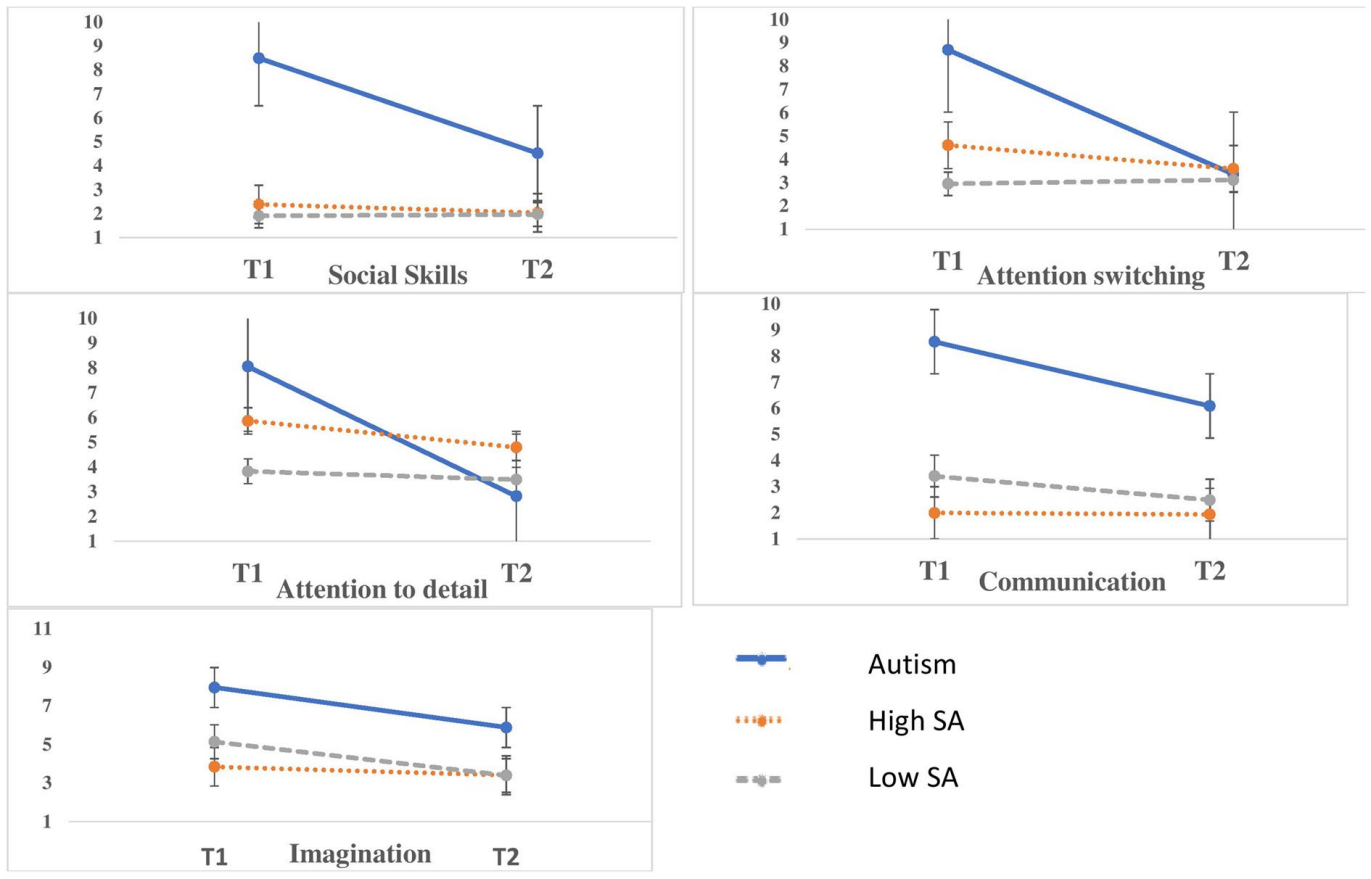
AQ subscales scores among the study’s groups at T1 (beginning of the academic year) and T2 (beginning of the consecutive academic year). SA, social anxiety.

Figure 2 presents AQ subscale scores at T1 and T2 for the study groups. Figure 1 presents the reduction in AQ scores from T1 to T2 for the study groups in standard deviations (norms for SD’s were taken from (1)).

Additionally, the 2 × 3 MANOVA for AQ subscales yielded a significant Time and a significant Group effect for all AQ subscales. However, the Time effect did not remain significant when using Age as a covariate and remained significant only for Attention Switching and Attention to Detail when using Sex as a covariate.

*Anxiety scores change*. A main effect of Time on anxiety scores was evident [*F*(2, 76) = 5.44, *p* < 0.05, *η^2^_p_* = 0.09]. Univariate analyses indicated a significant Time effect for both State Anxiety [*F*(1, 77) = 6.91, *p* = 0.01, *η^2^_p_* = 0.08] and Trait Anxiety [*F*(1, 77) = 7.08, *p* = 0.09, *η^2^_p_* = 0.08], demonstrating lower levels at T2 (*M* = 36.67, *SD* = 10.57; *M* = 38.41 *SD* = 9.60) compared to T1 (*M* = 40.11 *SD* = 9.05; *M* = 41.91 *SD* = 10.82) for both state and trait anxiety, respectively, among the entire sample. The reduction in STAI scores in standard deviations was 0.29 and 0.30 for State and Trait anxiety scores, respectively [norms taken from (61)]. No significant correlations were observed between STAI indices and AQ subscale score change among any of the study groups. Comparing the year-end GPAs between the groups revealed no significant differences (see Table 1).

### 3.2. AQ and academic performance

A correlation analysis between change in AQ subscale scores (T1-T2) and year-end GPA was conducted separately for each group (Table 4). Only for the Autism group was a significant negative association observed between GPA and a reduction in AQ Social Skills, Communication, and Imagination subscale scores, meaning that a larger decrease from T1 to T2 in these AQ subscale scores were associated with a lower GPA.

**Table 4:** Correlation between grade point average (GPA) at the end of first year and change in AQ subscales after 1 year of university attendance (T1-T2).

		AQ – social skills T1-T2	AQ – attention switching T1-T2	AQ- attention to detail T1-T2	AQ- communication T1-T2	AQ- imagination T1-T2
ASD	GPA	−0.31*	−0.02	0.01	−0.35*	−0.29*
	*n*	35	35	35	35	35
High SA	GPA	−0.03	0.07	0.19	0.19	−0.10
	*n*	21	21	21	21	21
Low SA	GPA	0.15	0.26	0.01	−0.14	−0.16
	*n*	27	27	27	27	27

Bold values in table represent *p* <0.05.

### 3.3. Predictive values of participant characteristics and baseline levels of psychiatric symptoms

Finally, we examined the predictive value of participant characteristics on change in the AQ subdomain scores (T1-T2) after one academic year. No multicollinearity was observed between the regression predictive variables (all VIF’s < 1.8). The regression analysis with T1-T2 AQ Communication subscale scores as the dependent variable is presented in Table 5. In the final model, explaining 27.8% of the variance, older age and an autism diagnosis were associated with greater reduction in AQ Communication scores (T1-T2).

**Table 5:** Linear regression analysis for AQ Communication subscale score.

Variable	*B*	SE	*β*	*R^2^*	Δ*R^2^*
Step 1					
Age	0.39	0.23	0.20	0.04	
Sex	0.07	0.77	0.10		
Step 2					
Age	**0.56**	**0.22**	0.29*	0.17*	0.13*
Sex	1.08	0.75	0.16		
Autism diagnosis	**1.64**	**0.51**	0.37**		
T1 LSAS	−0.18	0.26	−0.08		
Step 3				. 20*	0.02
Age	**0.57**	**0.22**	0.30*		
Sex	1.03	0.75	0.15		
Autism diagnosis	**1.98**	**0.63**	0.44**		
T1 LSAS	−0.06	0.27	−0.02		
T1 BDI-II	−0.30	0.28	0.13		
T1 Y-BOCS II Comp	−0.15	0.32	−0.07		
Step 4				0.28**	0.09**
Age	**0.67**	**0.21**	0.35*		
Sex	0.93	0.71	0.14		
Autism diagnosis	**2.56**	**0.64**	0.59**		
T1 LSAS	−0.02	0.26	−0.00		
T1 BDI-II	−0.34	0.27	−0.15		
T1 Y-BOCS-II Comp	0.41	0.36	0.20		
Autism diagnosis * T1 Y-BOCS-II Comp	**−1.67**	**0.59**	−0.48**		

T1 LSAS, Time 1 Liebowitz Social Anxiety Scale total score; T1 BDI–II, Time 1 Beck Depression Inventory II; T1 Y-BOCS-II Comp, Time 1 Yale–Brown Obsessive–Compulsive Scale II Compulsion score. *p < 0.05, **p < 0.01.

**Figure 3:**
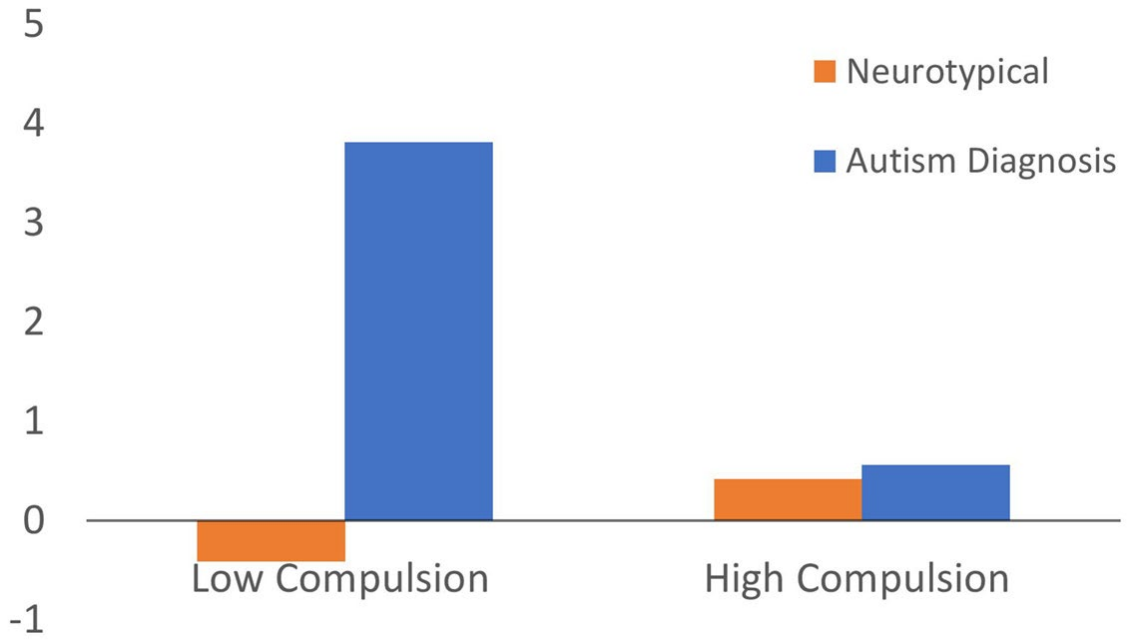
Interaction of autism diagnosis (YES/NO) and Compulsive symptoms (±1 SD from average) on the reduction in AQ communication score, according to regression equation.

Additionally, a T1 Y-BOCS-II Compulsion*Autism Diagnosis interaction was negatively associated with AQ Communication score decrease, such that a higher level of compulsive symptoms at T1 among autistic students was associated with a lesser reduction in AQ communication scores at T2. The interaction according to the regression equation is depicted in Figure 3.

In addition, two significant regression models were obtained for AQ Attention Switching (*R^2^* = 0.64 *p* < 0.001) and Attention to Detail (*R^2^* = 0.66, *p < 0.*001) subdomain scores as dependent variables. For both subscales in the final model, older age and an autism diagnosis were associated with greater reduction in these AQ subscale scores after one academic year at the university. No other significant models were observed.

## 4. Discussion

The main aim of this study was to explore whether there is change in autistic students’ self-reported autistic traits while attending university. We observed a robust decrease (over two standard deviations in three out of five AQ subscales) in autism trait levels as reported by the autistic students after attending university for one academic year. Autistic trait reporting is considered reliable and has demonstrated relative stability over time among autistic individuals as well as among the general population (1, 62–65).

While previous research has linked AQ scores and other mental factors such as anxiety (66, 67), depressive symptoms (36, 37), or mental distress (39), the reduction in autism traits found in this study cannot be explained by alteration in these factors, anxiety in particular. This conclusion is derived from the following study findings: (1) changes in trait and state anxiety did not correlate with any AQ subscale score change; (2) anxiety and depression levels at T1 were not associated with reduced AQ scores at T2; and (3) among autistic students, the decrease in their AQ scores (~2.0 *SD*) was far more striking than their reduction in state/trait anxiety (~0.30 *SD* in STAI scores).

Our finding of a robust reduction in autistic traits among students with autism could be explained by changes in cognitive factors, in particular attention. Among the Autism group (as well as the entire sample) the effect size for the change over time in the AQ Attention Switching subscale was the largest. Both the AQ Attention Switching and Attention to Detail subscales are measured through items that directly relate to attentional abilities (“In a social group I can easily keep track of several different people conversations”; “I often notice small sounds when others do not”). Thus, a possible interpretation of the robust autistic trait reduction is that it actually reflects change in cognitive abilities (attention in particular) expressed in the individual’s self-reported AQ score. Indeed, atypical attention patterns are common among autistic individuals (68) particularly in deploying attention from one stimulus to another (attention switching) (69–73). Accordingly, repeated elevated levels of social interaction afforded during 1 year of participating in the university’s integration program may have demanded more attention switching and were followed by improved attentional abilities, reflected in the students’ reports on the AQ. However, changes in other domains of autism traits were also reported by the students, perhaps indicating that change in the reported levels of autism traits cannot be explained solely by improved attention switching. Another plausible explanation relates to reduced meta-cognition in autism, particularly meta-cognition regarding self-representation (74): possibly, the autistic students’ self-perceptions of their level of autistic traits (as opposed to an objective assessment of their autism trait levels) simply changed following the intense social interactions inherent to attending university. However, the notion that the change reflects only a subjective, possibly biased perception is undermined by the finding of a significant association between autism trait reduction and GPA, a more objective measure of performance. Future studies should methodically and directly examine these possibilities, as they were not directly examined in this study.

The extensive reduction observed in the Communication subdomain of the AQ was associated with an autism diagnosis. However, this reduction was less profound among autistic students reporting high baseline levels of compulsion. The moderating effect of compulsion could be related to impaired extinction processes or, alternatively, to reduced social cognition, both previously associated with obsessive compulsive symptoms (75, 76).

As noted above, autistic students’ self-reported decrease in AQ scores was correlated with an objective measure, namely their grade point average at the end of the academic year. Previous studies have reported contrasting findings, with one study reporting a positive association (43) while another reporting a negative association (38) between autism traits and academic performance. Future studies should further examine these issues.

Although a statistically significant effect of time on autism traits was found for the ND groups, a close inspection of the findings suggests this reduction had small meaning in the clinical sense. According to Baron-Cohen et al. (1) the average AQ score for male student is 18.6 (SD 6.6) In this study, AQ scores reduced from 17.08 to 13.56 and from 19.20 to 15.68 from T1 to T2 in the Low and High social anxiety symptoms groups, respectively. This finding indicated the existence of small fluctuations in AQ scores among ND students, but this reduction was relatively small and within the normal range of AQ scores. For both ND groups, the AQ scores at both timepoints were within non-clinical range. Previous research has demonstrated such reductions and associated them with reduction in anxiety, among ND students (39). Future studies should examine changes in AQ scores among ND students.

This study, one of a small number of follow-up studies on university students with autism, has various strengths, including the participation of a relatively large group of university students with autism, a population with the highest potential for achieving independent living yet rarely examined in research. The use of standardized measures of psychopathology symptoms is an additional study strength. The novelty of this study includes its two control groups of ND students, and the comparison between autistic trait levels and self-reported psychopathology symptoms among university students over a relatively prolonged period (one academic year).

Notwithstanding the novel findings, the study has limitations. These include the infeasibility of a control group of autistic students not participating in the university’s integration program, as all students identifying as autistic chose to participate in it. Accordingly, it is difficult to isolate the major factor affecting the decrease in autism traits among the autistic students. Thus, to a certain extent, the generalizability of the findings may compromised, making them more applicable to autistic students participating in a university support/transition programs. Future studies should compare autistic students participating in support/transition programs to those who do not, and to those attending alternative post-secondary vocational or academic pursuits. Additionally, since it is also possible that behavioral changes (more social interaction) may underlie the reduction in autism traits, future studies are advised to monitor such changes. Another study limitation relates to the fact that measures of autism traits, anxiety, and depression were obtained by self-report questionnaires; diagnostic interviews conducted by mental health professionals are preferable for future studies. Further, participants in the high social anxiety group demonstrated what may be considered moderate levels of social anxiety symptoms and future studies are encouraged to study participants with even higher levels of social anxiety symptoms. Lastly, the male to female ratio of 39:2 among the autistic students is higher than the typically reported 4:1 male to female ratio in autism (77, 78), so future studies should attempt to include higher rates of females and achieve more balanced male/female ratios in all study groups.

The current study findings have important theoretical and clinical implications. Previous autism research and clinical effort reporting improvement in outcomes among toddlers was interpreted as due to increased brain plasticity during early development (79, 80). However, the current finding suggests the possibility of robust change during the young adulthood of autistic individuals too. The potentially dramatic lifestyle changes evoked by entering post-secondary education may lead to greater mental health problems (81) but simultaneously may also facilitate processes that accelerate acclimation and improvement in core symptoms. Our findings suggest that autism traits, as measured by the AQ, may be more malleable than conventionally thought. Further studies are needed to better characterize possible alterations in autistic traits over time, as an expression of the broader autistic phenotype, when autistic individuals transition to university and generally over their lifetime.

## Data availability statement

The raw data supporting the conclusions of this article will be made available by the authors, without undue reservation.

## Ethics statement

The studies involving human participants were reviewed and approved by The University of Ariel Ethics Committee. The patients/participants provided their written informed consent to participate in this study.

## Author contributions

All authors listed have made a substantial, direct, and intellectual contribution to the work and approved it for publication.

## Conflict of interest

The authors declare that the research was conducted in the absence of any commercial or financial relationships that could be construed as a potential conflict of interest.

## Publisher’s note

All claims expressed in this article are solely those of the authors and do not necessarily represent those of their affiliated organizations, or those of the publisher, the editors and the reviewers. Any product that may be evaluated in this article, or claim that may be made by its manufacturer, is not guaranteed or endorsed by the publisher.
